# A novel model of double replications and random loss accounts for rearrangements in the Mitogenome of *Samariscus latus* (Teleostei: Pleuronectiformes)

**DOI:** 10.1186/1471-2164-15-352

**Published:** 2014-05-09

**Authors:** Wei Shi, Xian-Guang Miao, Xiao-Yu Kong

**Affiliations:** CAS Key Laboratory of Tropical Marine Bio-resources and Ecology, South China Sea Institute of Oceanology, Chinese Academy of Sciences, 164 West Xingang Road, Guangzhou, 510301 People’s Republic of China

**Keywords:** Flatfish, Flounder, Mitochondrial recombination, Gene order, Molecular rvolution

## Abstract

**Background:**

Although more than one thousand complete mitochondrial DNA (mtDNA) sequences have been determined in teleostean fishes, only a few gene rearrangements have been observed, and genome-scale rearrangements are even rarer. However, flatfishes (Pleuronectiformes) have been identified as having diverse types of mitochondrial gene rearrangements. It has been reported that tongue soles and the blue flounder mitogenomes exhibit different types of large-scale gene rearrangements.

**Results:**

In the present study, the complete mitochondrial genome of another flatfish, *Samariscus latus*, was sequenced, and genome-scale rearrangements were observed. The genomic features of this flounder are different from those of any other studied vertebrates, including flatfish species too. The mitogenome of *S. latus* is characterized by the duplication and translocation of the control region (CR). The genes located between the two CRs are divided into two clusters in which their relative orders are maintained.

**Conclusions:**

We propose a “Double Replications and Random Loss” model to explain the rearrangement events in *S. latus* mitogenome. This model consists of the following steps. First, the CR was duplicated and translocated. Subsequently, double replications of the mitogenome were successively initiated from the two CRs, leading to the duplication of the genes between the two CRs. Finally, one of each pair of duplicated genes was lost in a random event.

**Electronic supplementary material:**

The online version of this article (doi:10.1186/1471-2164-15-352) contains supplementary material, which is available to authorized users.

## Background

The vertebrate mitochondrial genome typically codes for 37 genes, including 13 protein-coding genes, 22 transfer RNAs (tRNAs) and two ribosomal RNAs (rRNAs). These genes are arranged on a highly compact circular genome [1–3]. The order of the genes was initially considered conserved among vertebrates. However, with more than 1700 complete mitochondrial DNA (mtDNA) sequences currently determined, mtDNA gene rearrangements have been found in several groups, including birds [4–6], reptiles [7, 8], amphibians [9, 10] and fishes [11–13].

Gene rearrangements in animal mtDNA can be explained using four available models. (1) The Recombination model, which is characterized by the breakage and rejoining of the participating DNA strands [14], has been used to account for changes in the mitochondrial gene order in frogs, birds, mussels and other organisms [15–17]. (2) Another commonly accepted hypothesis is the Tandem Duplication and Random Loss (TDRL) model, which posits that rearrangements of the mitochondrial gene order have occurred via tandem duplications of some genes, followed by the random deletion of some of the duplications [18, 19]. This model is widely used to explain gene rearrangements in vertebrate mtDNA [4, 11, 20, 21]. (3) The other two models are seldom used; one is the Tandem Duplication and Non-Random Loss (TDNL) model proposed by Lavrov et al. [22], and the other is the tRNA miss-priming model [23, 24].

Fishes constitute the vertebrate group with the largest number of determined complete mtDNA sequences (1107 species as of July 2013; http://www.ncbi.nlm.nih.gov/genomes). However, only a few gene rearrangements have been observed in fish [12, 13, 25–27], and genome-scale rearrangements are rare [11]. Flatfish (Pleuronectiformes) have been found to be a diversified group for mitogenome rearrangements. Studies of the *Cynoglossus semilaevis* and *Paraplagusia japonica* (tongue soles, Cynoglossidae) mitogenomes have discovered the translocation of their control regions and a tRNA gene inversion [12]. However, no gene rearrangements have been detected in soles (Soleidae), the closest family to the Cynoglossidae [28–30]. This interesting event has also occurred in two other species belonging to close families Bothidae and Paralichthodidae. The mitogenome of *Crossorhombus azureus* (blue flounder) exhibits genome-scale gene rearrangements, whereas the gene order of *Pseudorhombus cinnamoneus* is intact [31]. These findings imply that the origin of gene rearrangements in different groups of Pleuronectiformes was likely a case of “polyphyly”. Due to the short divergence time between the fishes with and without gene rearrangements, many residual traces of these rearrangements may have been preserved; the mechanism of the rearrangement events may be inferred from these traces. Kong et al. [12] developed a model of inverse duplication and the deletion of redundant genes to explain gene rearrangements in tongue soles. To account for the gene rearrangements in blue flounder, Shi et al. [31] advanced the Dimer-Mitogenome and Non-Random Loss model (DMNR) that inferred the course of gene rearrangement in the blue flounder based on the typical gene order.

In the present study, the complete mitogenome of the deep-body righteye flounder, *Samariscus latus*, was sequenced, revealing genome-scale rearrangements. The gene order of this flounder is different from those of tongue soles, blue flounder and all other vertebrate species reported so far and none of the gene rearrangement models can account for this event properly. Thus, we deduced a novel mechanism of gene rearrangement for this flounder’s mitogenome.

## Methods

### Ethics statement

The fish specimen used in the present study was marine captured and purchased from Dasi seafood market in Keelung, Taiwan. The species was not involved in the endangered list of IUCN (http://fishdb.sinica.edu.tw/chi/species.php?id=382768). Specimen collection and maintenance were performed in strict accordance with the recommendations of Animal Care Quality Assurance in Taiwan and Chinese Mainland.

### Sampling, DNA extraction, PCR and sequencing

The specimen of *S. latus* was collected from Taiwan. A portion of the epaxial musculature was excised from fresh specimen and was immediately stored at -70°C. Total genomic DNA was extracted using the SQ Tissue DNA Kit (OMEGA) following the manufacturer’s protocol. Based on alignments and comparisons of complete mitochondrial sequences of flatfishes, dozens of primer pairs were designed for the amplification of the mtDNA genomes (Additional file 1: Table S1). More than 30 bp of overlapping fragments between tandem regions were used to ensure the correct assembly and integrity of the complete sequence.

PCR was performed in a 25-μl reaction volume containing 2.0 mM MgCl_2_, 0.4 mM of each dNTP, 0.5 μM of each primer, 1.0 U of Taq polymerase (Takara, China), 2.5 μl of 10x Taq buffer, and approximately 50 ng of DNA template. The PCR cycling conditions included an initial denaturation at 95°C for 3 min followed by 30–35 cycles of denaturation at 94°C for 45 s, annealing at 45–55°C for 45 s, and elongation at 68–72°C for 1.5-5 min. The reactions were completed by a final extension at 72°C for 5 min. The PCR products were purified with the Takara Agarose Gel DNA Purification Kit (Takara, China) and were used directly as templates for cycle sequencing reactions. Sequence-specific walking primers were designed for both strands of each fragment and were employed for sequencing with an ABI 3730 DNA sequencer (Applied Biosystems, USA). The mtDNA sequences of *S. latus* have been submitted to GenBank under the accession number KF494223.

### Sequence analysis

The sequenced fragments were assembled to create complete mitochondrial genome using CodonCode Aligner v3 and BioEdit v7 [32]. During the processing of large fragments and walking sequences, regular manual examinations were performed to ensure the reliable assembly of the genome sequence. The annotation and boundary determination of protein-coding and ribosomal RNA genes were performed using NCBI-BLAST (http://blast.ncbi.nlm.nih.gov). Transfer RNA genes and their secondary structures were identified using tRNAscan-SE 1.21 [33], with the cut-off values set to 1 when necessary.

## Results and discussion

### Novel gene order in *S. latus* mitogenome

**Figure 1:**
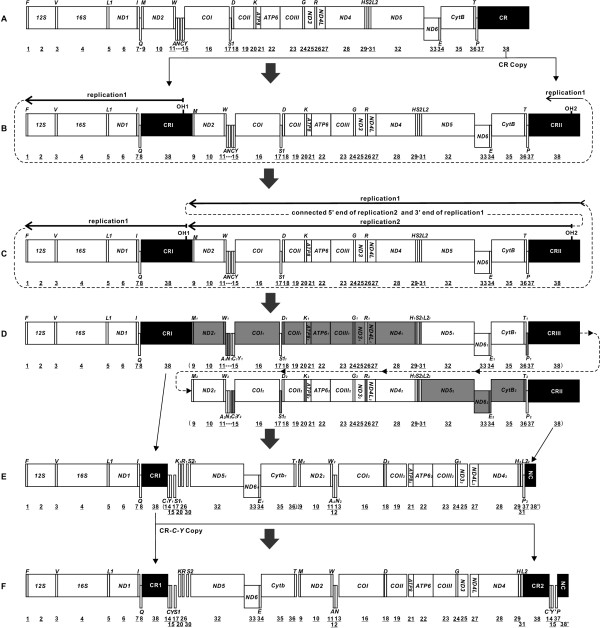
**The proposed mechanism of the rearrangement of the ancestral gene order to that of** ***S. latus*** **mitogenome.** Protein-coding genes and CRs are indicated by boxes, and tRNA genes are indicated by columns. Genes labeled above the diagram are encoded by the H-strand and the others by the L-strand. OH indicates the origin of replication for the H-strand; the direction of replication is shown by arrows. Consecutive numbers (1–38) indicate the gene order. The dark boxes indicate the control regions (CRs) and noncoding region (NC). **(A)** The ancestral mitogenome with 37 genes and one CR. **(B)** The ancestral CR was duplicated to CRI and CRII, and then CRI translocated to the position between *tRNA-Q* and *tRNA-M*. A mitochondrial replication event (RP1) was initiated at OH1. **(C)** After RP1 passed through OH2, replication2 (RP2) began at OH2. Both replications terminated close to OH1 (namely, the 5′ end of the nascent strand of RP1). **(D)** Double replications led to the duplication of one CR and 29 genes. One of each copied gene pair was lost randomly. Dark gray boxes indicate the degenerated genes. **(E)** The gene order after the loss of several genes and CRs. **(F)** The gene order of *S. latus* mitogenome that was formed after CR and *tRNA-C-Y* duplicated and translocated to the position between *tRNA-L2* and *tRNA-P*.

The complete mitogenome of *S. latus* is 18,706 bp in length. The genome includes two rRNA genes, 24 tRNA genes, 13 protein-coding genes, two control regions (CR1 and CR2) and one 376-bp noncoding region (NC). Most of the genes are encoded on the H-strand, except for *ND6* and ten of the tRNA genes (including the copied *tRNA-C* and -*Y*) (Table 1, Figure 1).

**Table 1:** **Organization of** ***S. latus*** **mitochondrial genome**

	From	To	Size		Anticodon	Start	Stop	Strand	Intergenic
			bp	aa		Codon	Codon		Region
*tRNA* ^*Phe*^ (*F*)	1	68	68		GAA			H	0
*12 s rRNA*	69	1020	952					H	0
*tRNA* ^*Val*^ (*V*)	1021	1092	72		TAC			H	0
*16 s rRNA*	1093	2805	1713					H	0
*tRNA* ^*Leu1*^ (*L1*)	2806	2879	74		TAA			H	0
*ND1*	2880	3854	975	325		ATG	TAA	H	3
*tRNA* ^*Ile*^ (*I*)	3858	3927	70		GAT			H	-2
*tRNA* ^*Gln*^ (*Q*)	3926	3997	72		TTG			L	0
CR1	3998	4890	893					H	1
*tRNA* ^*Cys*^ (*C*)	4892	4958	67		GCA			L	0
*tRNA* ^*Tyr*^ (*Y*)	4959	5026	68		GTA			L	80
*tRNA* ^*Ser1*^ (*S1*)	5107	5177	71		TGA			L	85
*tRNA* ^*Lys*^ (*K*)	5263	5337	75		TTT			H	43
*tRNA* ^*Arg*^ (*R*)	5381	5450	70		TCG			H	88
*tRNA* ^*Ser2*^ (*S2*)	5539	5605	67		GCT			H	75
*ND5*	5681	7519	1839	613		ATG	TAG	H	-4
*ND6*	7516	8037	522	174		ATG	TAG	L	0
*tRNA* ^*Glu*^ (*E*)	8038	8106	69		TTC			L	4
*CYTB*	8111	9251	1141	380		ATG	T	H	0
*tRNA* ^*Thr*^ (*T*)	9252	9321	70		TGT			H	86
*tRNA* ^*Met*^ (*M*)	9408	9476	69		CAT			H	0
*ND2*	9477	10521	1045	348		ATG	T	H	0
*tRNA* ^*Trp*^ (*W*)	10522	10591	70		TCA			H	1
*tRNA* ^*Ala*^ (*A*)	10593	10660	68		TGC			L	1
*tRNA* ^*Asn*^ (*N*)	10662	10734	73		GTT			L	59
*COI*	10794	12344	1551	517			TAA	H	22
*tRNA* ^*Asp*^ (*D*)	12367	12434	68		GTC	GTG		H	5
*COII*	12440	13138	699	233		ATG	AGA	H	62
*ATP8*	13201	13368	168	56		ATG	TAA	H	-10
*ATP6*	13359	14042	684	228		ATG	TAA	H	-1
*COIII*	14042	14827	786	262		ATG	TAA	H	-1
*tRNA* ^*Gly*^ (*G*)	14827	14896	70		TCC			H	0
*ND3*	14897	15247	351	117		ATG	TAA	H	70
*ND4L*	15318	15614	297	99		ATG	TAA	H	-7
*ND4*	15608	16981	1374	458		ATG	AGA	H	7
*tRNA* ^*His*^ (*H*)	16989	17057	69		GTG			H	16
*tRNA* ^*Leu2*^ (*L2*)	17074	17147	74		TAG			H	0
CR2	17148	18046	899					H	0
*tRNA* ^*Cys’*^ (*C’*)	18047	18113	67		GCA			L	0
*tRNA* ^*Tyr’*^ (*Y’*)	18114	18181	68		GTA			L	78
*tRNA* ^*pro*^ (*P*)	18260	18330	71		TGG			L	0
NC	18331	18706	376					H	0

CR1, CR2: control region 1 and control region 2. NC: noncoding region. *tRNA* ^*Leu*1^: *tRNA* ^*Leu*^ *(TAA)*; *tRNA* ^*Leu*2^: *tRNA* ^*Leu*^ *(TAG)*; *tRNA* ^*Ser*1^: *tRNA* ^*Ser*^ *(TGA)*; *tRNA* ^*Ser*2^: *tRNA* ^*Ser*^ *(GCT)*; *tRNA* ^*Cys’*^ (*C’*), *tRNA* ^*Tyr’*^ (*Y’*): a copy of *tRNA* ^*Cys*^ and *tRNA* ^*Tyr*^. Intergenic spacers are located between the feature on the same line and that on the following line; a negative number indicates an overlap.

The genome-scale mitogenome rearrangements in *S. latus* make the mitogenome of this species differ greatly from other known vertebrate mitogenomes. The typical gene order of vertebrate mitogenomes and that of *S. latus* mitogenome are shown in Figure 1A and F, respectively. The genes of the typical genome are numbered consecutively from 1 (*tRNA-F*) to 38 (CR) (Figure 1A). Compared with the typical mitogenome, that of *S. latus* has undergone extensive changes to produce the following novel order: 1, 2, 3, 4, 5, 6, 7, 8, *38, (14, 15*, 17, 20, 26, 30, 32, 33, 34, 35, 36), (9, 10, 11, 12, 13, 16, 18, 19, 21, 22, 23, 24, 25, 27, 28, 29, 31), *38, 14, 15*, 37 and 38′ (Figure 1F). The rearrangement of *S. latus* mitogenome exhibits the following unique features: The first eight genes (1–8) retain the original position and order, followed by the insertion of a CR (38, designated CR1). The typical order (9–36) is largely changed into two gene clusters: an 11-gene cluster (14, 15, 17,…36) and a 17-gene cluster (9, 10,…16…31), in both clusters the typical relative orders of the genes are maintained (from small to large). The other CR*-C*-*Y* fragment follows (designated CR2, *C’*, *Y’*). The site of the last tRNA gene, *tRNA-P* (37), is unchanged, and the end is a noncoding fragment (NC, designated 38′).

### Intergenic regions and control regions

The *S. latus* mitogenome contains 19 intergenic regions, nine of which are over 50 bp in length (an uncommon occurrence). Given the parsimonious nature of the vertebrate mitogenome, the presence of so many long intergenic regions is surprising. We hypothesize that the reason for this phenomenon is that the intergenic regions are likely to be the residual sequences of genes that degenerated during the process of gene rearrangement. Moreover, three large noncoding regions of CR1, CR2 and NC are longer than 300 bp. The NC is located at the typical CR position, between the *tRNA-F* and *tRNA-P* genes, but it shows no sequence similarity to CRs in other flatfishes. Two CR fragments, CR1-*C*-*Y* (between *tRNA-Q* (8) and *tRNA-S1* (17)) and CR2-*C’*-*Y’* (between *tRNA-L2* (31) and *tRNA-P* (37)), are identical in sequence. Compared with the sequences of other flatfish CRs, CR1 and CR2 exhibit the typical tripartite structures of the CR: terminal associated sequences (TAS), central conserved sequence blocks (CSB-D, E), a pyrimidine tract (PY region) and conserved sequence blocks (CSB-2, 3) [34–37] (Additional file 2: Figure S1). The structures of CR1 and CR2 support the opinion that these two fragments are indeed the control regions same as other fishes except that *S. latus* mitogenome possesses two identical control regions.

### Gene rearrangement mechanism of *S. latus* mitogenome

Mitogenome rearrangements have been reported in flatfishes (two tongue soles and blue flounder) [12, 27, 31]. The rearrangement of tongue sole mitogenomes is characterized by tRNA gene inversion, whereas that of the blue flounder is characterized by an L-strand coding gene cluster. However, the characteristics of *S. latus* rearrangements are completely different from those of the above flatfishes; therefore, there is little possibility that the gene order of *S. latus* mitogenome was derived from that of these flatfishes. Thus, the models mentioned above [12, 31] that explain the mtDNA rearrangements in these flatfishes do not perfectly suit to *S. latus* mitogenome.

Some models, such as recombination [14] and TDRL [18, 19], might help explain the mechanism of *S. latus* mitogenome rearrangements. However, several unique features of *S. latus* rearrangements prevent the application of these models to this species. For example, the recombination model requires that several (more than five) recombination events occurred during the course of rearrangement. Moreover, gene rearrangements caused by recombination are unusual in the teleost fishes. The Tandem Duplication and Random Loss Model (TDRL) might have the applicability for gene rearrangements in *S. latus* mitogenome [18, 19], however, this model fails to highlight the specific features of *S. latus* mitogenome, such as gene clusters separated by CRs and its remnants. As for the tRNA miss-priming [23, 24] and TDNL models [22], no obvious corresponding characteristics exist in *S. latus* mitogenome.

**Figure 2:**
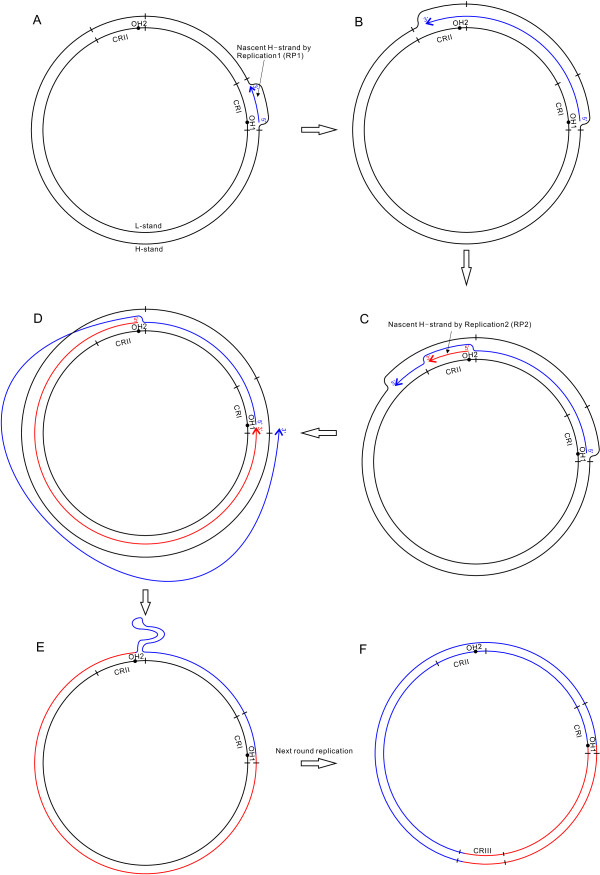
**A duplication caused by double replications. (A)** A mitochondrial replication event (RP1) was initiated at OH1. **(B)** RP1 passed through OH2. **(C)** RP2 was initiated at OH2. **(D)** Both RP1 and RP2 terminated at OH1, while the nascent H-strand of RP1 was replaced by that of RP2. **(E)** The connection of the 3′ end of RP2 to the 5′ end of RP1, and the connection of the 5′ end of RP2 to the 3′ end of RP1. **(F)** The duplication was made permanent in the next round of replication.

The ancestral gene order of *S. latus* mitogenome is most likely the typical vertebrate order because the mtDNA of all other flatfishes (more than ten species) follows the typical order. Therefore, we propose a “Double Replications and Random Loss (DRRL)” model to describe the rearrangement events that changed the ancestral gene order to that observed in *S. latus* mitogenome (Figure 1A). The hypothesized intermediate steps from a typical gene order to that of *S. latus* gene arrangement are as follows. First of all, the CR was duplicated to CRI and CRII, and then the CRI was translocated into the *IQM* (7–9) tRNA gene cluster. These events produced a genome with two CRs (CRI and CRII), both of which contained origin of heavy (OH) strand replication (OH1 and OH2) and either of which could serve as the mitochondrial origin of replication (Figure 1B). Second, mitochondrial replication (replication1, RP1) was first initiated at OH1 (Figure 1B, Figure 2A). Subsequently, after the replication elongation passed through OH2 (Figure 2B), the other replication (replication2, RP2) began at OH2 (Figure 1C, Figure 2C). Both replications terminated at OH1 (the 5′ end of the nascent strand of RP1; Figure 1C, Figure 2D). The closure event of the circular mtDNA connected the 5′ end of the nascent strand of RP1 to the 3′ end of RP2. The connection of the 5′ end of RP2 to the 3′ end of RP1 could be completed by another circular closure event or by an mtDNA repair mechanism afterwards (Figure 1C, Figure 2E). The double replications resulted in a copy of 29 genes (indicated by subscript 1 & 2 to the letters) from *tRNA-M* (9) to *tRNA-P* (37), with three CRs (CRI, CRII and CRIII) in the mtDNA (Figure 1D; Figure 2F). In subsequent evolutionary events, one of each of the 29 duplicated gene pairs was randomly lost (Figure 1D; gray boxes). The three CRs followed different courses: CRI retained its function, while CRII and CRIII degenerated into two noncoding regions: NC (38′) and an 86-bp intergenic region between *tRNA-T* (36) and *tRNA-M* (9) (Figure 1E). Finally, the fragment of CRI- *C*_*1*_*-Y*_*1*_ (Figure 1E) were duplicated once, which were respectively named CR1-*C-Y* and CR2*-C’*-*Y’*, followed by the latter translocated to a position between *tRNA-L2* (31) and *tRNA-P* (37) (Figure 1F). These genome-scale rearrangements resulted in the mitogenome of *S. latus* containing two CRs, 24 tRNAs, one 376-bp noncoding region and several long intergenic regions (Figure 1F, Table 1).

### Details of the model

The evidence supporting the rearrangement event in the final step of the proposed model (Figure 1E-F) is the presence of completely identical sequences in CR1-*C*-*Y* and CR2-*C’*-*Y’*. This finding indicates that both fragments were derived from the same original template during a very recent duplication and translocation event. Based on tRNA-*C, Y* is located between *tRNA-Q* and *tRNA-S1* in the typical vertebrate mitogenome, we infer that CR1-*C*-*Y* is CRI- *C*_*1*_*-Y*_*1*_ at the original template location (Figure 1E) and CR2-*C’*-*Y’* is a copy that then translocated to the site between *tRNA-L2* and *tRNA-P*. More specifically, the recent ancestral gene order for *S. latus* mitogenome (Figure 1F) is 1, 2, 3, 4, 5, 6, 7, 8, 38, (14, 15, 17, 20, 26, 30, 32, 33, 34, 35, 36), (9, 10, 11, 12, 13, 16, 18, 19, 21, 22, 23, 24, 25, 27, 28, 29, 31, 37, 38′), as illustrated in Figure 1E.

Both the copies of the original duplication of the CR (CRI and CRII, Figure 1A to B) and CR-*C-Y* (CRI- *C*_*1*_*-Y*_*1*_ and CR2-*C’*-*Y’*, Figure 1E to F) can be explained by TDRL or other possible mechanisms, however, there is no clear indication to confirm which one is more suitable for those duplication events. The duplication and translocation of CR is relatively common in gene-rearranged mitogenomes of metazoan, such as thrips, frogs, agamids, parrots, and so on [4, 10, 38, 39], which also occurred in teleost fishes [11].

The gene order depicted in Figure 1E clearly indicates that the genes from 9 to 38 were divided into two clusters (14, 15, 17,…36 and 9, 10,…16…38′), each of which retained the conserved relative gene order, from small to large. It is reasonable to assume that the two clusters were derived from a duplicated DNA fragment that spanned genes 9 to 38 in the typical gene order (Figure 1D). A remarkable characteristic of the duplicated DNA fragment depicted in Figure 1D is that it begins at one CR (CRI in Figure 1C) and ends at another CR (CRII in Figure 1C).

Based on the characteristics of gene clusters separated by non-coding regions and CRs, we propose the “Double Replications” model to explain the duplication of the DNA fragment spanning genes 9 to 38 in the typical gene order. There are three distinctive differences between the proposed “Double Replications” process (Figure 2) and normal mitochondrial replication. One difference is the origination of the double replications from two CRs (CRI and CRII) in one mitogenome (Figure 2A-C). This is a possible process because there have been similar events in dimeric mitogenomes (two monomeric mitogenomes linked head-to-tail) [40]. Clayton has studied these kind of events and detailed the process of “Double Replications” in unicircular dimeric mouse mtDNA with double CRs. Second, both replications (RP1 and RP2) terminated at OH1; after originating from OH1, RP1 did not terminate at the first encountered OH (OH2) (Figure 2D). The reason for this phenomenon is as follows: When RP1 proceeded to OH2, RP2 did not begin, and the relevant proteins or RNA primer did not bind to OH2 (Figure 2B). Therefore, there was no termination signal [40, 41], and RP1 could bypass this region and normally terminated at OH1, where the proteins and RNA primer were bound when RP1 started. RP2 also terminated at OH1 because the first termination signal encountered by RP2 was also the one at OH1 (Figure 2D). Finally, the circular closure event of the double replications was different from normal replication, which the four ends (the 3′ and 5′ ends of the nascent H-strands of RP1 and RP2) needed to be closed in pairs (Figure 2D). Because the initiation of RP2 occurred later than that of RP1, the nascent H-strand of RP1 would have been replaced by that of RP2 from CR II to CRI. As a result, only the 5′ end of RP1 and the 3′ end of RP2 would remain bound at CRI on the template L-strand, and the simplest means of connecting the ends would be the circular closure event at CRI (Figure 2D). The remaining two ends, the 5′ end of RP2 bound to CRII and the freed 3′ end of RP1, could be joined by the other circular closure event at CRII or by mitochondrial repair [42] (Figure 2E). These double replications would represent a feature specific to triple CRs (Figure 1D, Figure 2F). As in *S. latus* mitogenome, the three CRs (CRI, CRII and CRIII) developed into fully functional CRs (CR1 and CR2), a degenerated CR (NC), and an 86-bp intergenic region between *tRNA-T* and *tRNA-M*, respectively; the latter has been almost entirely lost during evolution.

DRRL model is different from the TDRL by the following features. First are the fixed replication origin points (OHs) but not the random sites as in the TDRL; second is the duplicated region which should start from an OH and end at another OH. Indeed, the DRRL model still needs more experimental evidence to verify. Nonetheless, every step of the model follows the nature and rule of normal mitochondrial replications. And both replications (RP1 and RP2) in our model initiated from the normal initiation sites, and terminated at the first met R-loop (the double strands of DNA and RNA primer) sites [3, 43, 44]. Existing other models and explanations are not comparable on this point (No violate the nature and rule of mitochondrial replications).

## Conclusions

In summary, we determined the complete mitochondrial genome of a flatfish, *S. latus*. The genes of this mitogenome are extensively rearranged. The mitogenome is characterized by the duplication and translocation of the control region. The genes located between the two CRs are divided into two clusters in which their relative orders are maintained. We proposed a “Double Replications and Random Loss” model to explain the rearrangement events in *S. latus* mitogenome. First of all, the CR was duplicated and translocated. Double replications of the mitogenome were successively initiated from the two CRs, leading to the duplication of the genes between the two CRs. Lastly, one of each pair of duplicated genes was lost in a random event.

### Data accessibility

DNA sequences: Genbank accessions KF494223.

## Electronic supplementary material

Additional file 1: Table S1: The primers used for fragment amplification in *S. Latus* flatfish mitogenomes. (DOCX 14 KB)

Additional file 2: Figure S2: Aligned sequences of nine flatfishes CRs and the two CRs of *S. Latus.* (DOCX 31 KB)
