# Supplementary material for: A novel model of double replications and random loss accounts for rearrangements in the Mitogenome of Samariscus latus (Teleostei: Pleuronectiformes)
Source: BMC Genomics. 2014 May 9;15(1):352. doi: 10.1186/1471-2164-15-352 (PMC4035078; doi:10.1186/1471-2164-15-352)
Supplement: Supplementary file 1 — Additional file 1: Table S1: The primers used for fragment amplification in S. Latus flatfish mitogenomes. (DOCX 14 KB) [file 12864_2013_6040_MOESM1_ESM.docx]

Table S1 The primers used for fragment amplification in *Samariscus Latus* flatfish mitogenomes

| Forward  primer | Sequences (5′-3′) |  | Reverse  primer | Sequences (5′-3′) |
| --- | --- | --- | --- | --- |
| Z15 | ATTAAAGCATAACHCTGAAGATGTTAAGAT |  | F49 | GGCCCATCTTAACATCTTC |
| Z2625 | GTTTACGACCTCGATGTTGGATCAGGACAT |  | F2671 | AGATAGAAACTGACCTGGAT |
| Z6468 | CCACATCTDCTGCATGCAAAYCAYACACTT |  | F2753 | TAGATAGAAACTGACCTGGATTACTCCGGT |
| R8010 | CCMCGACGCTACTCTGACTA |  | F5196 | CTAAATGGTTGGGGTATGG |
| Z10818 | TTYGAAGCAGCCGCMTGATACTGACAYTT |  | F6746 | GCGGTGGATTGTAGACCCATARACAGAGGT |
| Z13347 | AAGGATAACAGCTCATCCGTTGGTCTTAGG |  | F11089 | TTTAACCAAGACCRGGTGATTGGAAGTC |
| Z13916 | TGRGARGGTGTAGGHATTAT |  | F13413 | TAGCTGCTACTCGGATTTGCACCAAGAGT |
| L14734 | AACCACCGTTGTTATTCAACT |  | H15149 | CTCAGAATGACATTTGTCCTCA |
| L17114 | RCGCCCAAAGCTAGDATTC |  | F17147 | TAGTTTARTGCGAGAATCCTAGCTTTGGG |
