# Supplementary material for: A novel model of double replications and random loss accounts for rearrangements in the Mitogenome of Samariscus latus (Teleostei: Pleuronectiformes)
Source: BMC Genomics. 2014 May 9;15(1):352. doi: 10.1186/1471-2164-15-352 (PMC4035078; doi:10.1186/1471-2164-15-352)
Supplement: Supplementary file 2 — Additional file 2: Figure S2: Aligned sequences of nine flatfishes CRs and the two CRs of S. Latus. (DOCX 31 KB) [file 12864_2013_6040_MOESM2_ESM.docx]

Figure S1 Aligned sequences of nine flatfishes CRs and the two CRs of *Samariscus Latus.*

TAS-1

CR1 TATATACATA TATATGTATA ATCACCATTA ATTTATATTA ACCATTTCTT AGGATGTAAA TTAGCATATT TTGATATATA ACCTGTTATT AAATTAAACC AATCATAAGC AATCGAAGAT [120]

CR2 .......... .......... .......... .......... .......... .......... .......... .......... .......... .......... .......... .......... [120]

Hhi .TC...T.C. .G......AT .A......AT .......G.. .......TA. GT......CT AGGA....CA .GT...-..C ...AAA.C.C GT.A..C.G. .C....TCAT C.A.--.TT. [120]

Hst .TC...T.C. .G......AT .A......AT .......G.. .......TA. GTA.....CT AGGA....CA .GT...-..C ...ACA.C.C GT.A..G.A. .C....TCA. C.C.--.TT. [120]

Rhi .T....T.C. .G.......T .A......AT .......G.. .......TG. GTA.....CT AGGA....CA .GT...-..C ....AAC..A GT.A..T.G. .C....TCAT C.C.--.TT. [120]

Vmo .T...GT.C. ........AT .A......AT .......G.. ........A. GT......TC AGGA...TCA .GT...-... ....AA.C.A GT....T.G. .C....TCAT C.A.--.TT. [120]

Vva .T...GTGC. ........AT .A......AT .......G.. .......TA. GT......CT AAGA...TCA .GT..T-... ....AA.C.A GT.A..T.G. .C....TCA. C.A.--.TT. [120]

PCo .T....G.C. ........AT TA......AT .......G.. .......TA. .CAG....TC GGGA...T.A .GT.AT-..C .G..A.A.CA GTT..T..A. ..A...G-CT CGGT---A.. [120]

Pst .TC..GT.C. .G......AT .A......A- -------G.. .......TA. .TA...A.CT AGGA...TCA .GT...-... ....AA.C.A GT.A..T.G. .C....TCAA C.A.--.TT. [120]

Pol .TA...T.C. ........AT TA......AT .......G.. .A....AAG. CC......C- AAGA..C.AA .G...G-TG. ..AAAAC..G GTG.C.-.A. .T.....TA. C.G.---T.. [120]

Pma .T..-.T.C. .........T .A........ ..C....... .......AGA CC.G...CCG .C.A...TAG .GAT..-..C .GTAAAGTA. GG.G.T..A. .T...AG-AT C.A.---T.C [120]

CR1 TCAGCTCAAA TTG---AAAA ATC-CAACGA TTTGTTTAGT TTACTCA--- AACATTTCAA TTTTAATATA AACCTCCAAC CAAGACATGT GACGTATCT- TGACATTCGA CTAATACTC- [240]

CR2 .......... ...---.... ...-...... .......... .......--- .......... .......... .......... .......... .........- .......... .........- [240]

Hhi .T.A..A.G. .A.-ACT... .C.-TG.AT. A.CAC.A.TC ...AAT.TGT G.A.G.C..G GACC.G.CG. ..T..AAG.. .G.-...CAA C..TC...AG .CGAG..AT. .C..G....A [240]

Hst .T.A..A.G. CGA-ACT... .C.-TG.GTG A.CAC.A.TC ...AAT.AGT G.A.G.C..G GACC.G.CG. G.T..AAG.. .G.-...CAA C..TC...AG .CGAG..AT. .C..G....A [240]

Rhi .T.A..A.G. AATTACT.G. .C.CTG..TT CACA..C.CG ....AT.TGT G.A...C..G GACC.GCCG. C.TT.AAG.. .G.-...CAA C..TC.C.GG .C.AG..AT. .C..G....A [240]

Vmo .T.A.CA.GG .AA-ACT... .C.-T...T. A.CA..G.T. ...AAT.TAT G.A...C..G GAC..G.CG. ...T.AAG.. .G.-...C.A C..TC...AG .C.AG..AT. .C..G....A [240]

Vva .T.A..A..G .AA-ACT... .C.-T..TT. A.CAC.A.T. ...AAT.TGT ..A.C.CT.G GACC...CG. ..TT.AAG.. .G.-...C.A C..TC...AG .T.AG..AT. .C..G....A [240]

PCo ...CACT..G G.AGAC.... .C.--..... .-..AAA.TC G..AATTAAC .GA.-A.T.. GACC...CG. G.TT.AAG.. .T.-..TAA. T.TTC.C.AG .T.AGA.AT. .C..G....A [240]

Pst .T.A..A.G. .A.-ACT.G. .C.-...TCC CCCAC.G.CC ....ATTACT GGA.C.ATGG GGCC.GCCG. G.TT.AAG.. .G.-...CAA C..TC...AG .CGAG..AT. .C..G....A [240]

Pol AT.A..A..T A.ATAC.... .C.------. AACC.A..AG G..TA..ATA ..G.A..A.. GAC..G.CG. ...T.TAC.. .G.-...CAA CCTTC..A.G .C.AG..AT. .C..G....A [240]

Pma CGGA..TCT. GG.CAC.TG. ...--..AA. .ACAA.A.TC ACCA.G.TAC .GA.-.CG.G GGCA.G.CG. ..A.CAAG.T ...-.TCAA. T.TTC...CG .A.AG..AT. .CTT.....A [240]

CSB-E

CR1 ---------- ------CAAA ATTATGCGCA GTAAGAGCCG ACCAACCAGC TCATAACTTA AAGCTAACGG TTATTGATGG TCAGGGACAG ATATTGTGGG GGTTTCACCT CGTGAATTAT [360]

CR2 ---------- ------.... .......... .......... .......... .......... .......... .......... .......... .......... .......... .......... [360]

Hhi AAATCTCGCC GAC-CC.... ..CC.AT.T. .........T .......G.- .G..TC.... .T.A....TC .......G.. .G.......A .A........ ........TC G......... [360]

Hst AAATCTCGCC GAC-CT.... ...C.AT.T. .........T .......G.- .G..TC.... .T.A....TC .......G.. .G.......A .A........ ........TC G......... [360]

Rhi AAATTTCACT CAC-TTA... .ACCAA.C.. A........T .......G.- .G..CC.... .T.A....TC .......G.. .G.......A .A........ ........TC G......... [360]

Vmo AAATCTCGCC AACACA.... T.CC.AT.T. .........T .......G.- .G..TT.... .T.A....TC .......... .......... .A..C..... ........TC A.....C... [360]

Vva AAATCTCGCC CAT-CAT... T.CC.AT.T. .........T .......G.- .G..TT..G. .T.A....TC .......... .......... .A..C..... ........TC A.....C... [360]

PCo AAATCCCGAC ATT-TTG... .A...AT.T. .........T .......G.- .G..TC.... .T.A...... .......A.. .G.......A .A........ ........TC G......... [360]

Pst AAATCTC-TT CAC-AT..G. TCCC.AT.T. .........T .......G.- .G..T..... .T.A...... .......A.. .G.......A .A........ .......... .....T.... [360]

Pol AACTTCTGTC GAT-CC.... T.CCCAT... .........T ....T.AGT- .G..T..... .T..C..... .......A.. .G.......A .A........ ........A. A.....C... [360]

Pma CCATCCTATT GAC-CT...T CCCCGAT.T. ......A..T .....A.T.- .G..TT.... .T.A...... .......A.. .G.......A GA.CC..... ..CGG..... .T..C.C... [360]

CSB-D

CR1 TACTGGCATC TGGTTCCTAC TTCAGGGCCA TTAATTGCAA TT-TTCCCTA CACTTTTATC GACACTCGCA TAAGTTAATG GTGG-AAAGC ATATCTCACA AGTACCTTGC AAGCCGGGCC [480]

CR2 .......... .......... .......... .......... ..-....... .......... .......... .......... ....-..... .......... .......... .......... [480]

Hhi .C.......T .......... .......... ..G....ATG ..A.C..TC. ......C... ......TA.. .......... T..AT..TA. ...CGA.T.G T-....CAC. .........G [480]

Hst .C.......T .......... .......... ..G....AT. ..A.C..TC. ......C... ......TA.. .......... T..AT..TA. ...CGA.T.G T-....CA.. .........G [480]

Rhi .C.......T .......... .......... ..G....AT. ..A.C..TC. ......C... ......TA.. .......... T..AT..TA. ...CGA.T.G T-....CAC. .........G [480]

Vmo .C.......T .......... .......... ..G.C..AT. ..A.C..TC. ......C... ......T... .......... T..AT..TA. ...CGA.T.G T-....CA.. .........G [480]

Vva .C.......T .......... .......... ..T..C.AT. ..A.C..TC. ......C... ...G..T... .......... T..AT..TA. ...CGA.T.G T-....CA.. .........G [480]

PCo .C.......T .......... ......TT.T A...C..GT. .CA.C..TC. ......C... ......GA.. .......... T...T..TA. ...CGA.T.G T-....CAC. .........G [480]

Pst .C.......T .......... .......... .G...C.A-G .CAC....C. ......C... ...G..TA.. .......... T..AT..TA. ...CGA.T.G T-....CA.. .........G [480]

Pol .C.......T .......... .......... .G.CC..AT. ..A....TC. ......C... ......TA.. .......... T...T.TTA. ...CGA.T.G T-....CA.. .........G [480]

Pma .C.......T ...C...... ......T... ...CC..AT. ..A.....CC ......C... ...G..TA.. .......... ....A..C-. ...-ACTT.G T-....CCC. ......A..G [480]

PY region

CR1 TTCTCTCCAG GGGGTAAGGG GTTTTTTTTT ATTTTTTTCC TTTCACCTGG CATTTCACAG TGCACCC--C AAACCTAATT AAC--AGGAG CGTACATATT GCTTGCAC-C GGCCCCTAAA [600]

CR2 .......... .......... .......... .......... .......... .......... .......--. .......... ...--..... .......... ........-. .......... [600]

Hhi ...A...... C......... ...C.C.... -......... ......T..A .......A.. ....T..AG. C...GG.GAC GTTTA.A.G. T.AG..CT.. T.......G. .C.GTAC.T. [600]

Hst ...A...... C......... ...C.C.... -......... ......T..A .T.....A.. ....T..AG. C...AG.GAC GTTTA.A.G. G.AG..CT.. T.......G. .C.GTAC.T. [600]

Rhi ...A...... C......... ...C.C.... -......... ......T..A .......G.. ....T..AG. C...AG.GAC GTTTA.A.G. T.AG..CT.. T.......T. A..GTAC.T. [600]

Vmo ...A...... C......... ...C.C.... -......... .....T.... ....A..G.. ....T..AG. C..AAG..AC GTTTA.A.G. T.AG...T.. T.....T.G. ...GTAC.T. [600]

Vva ...A...... C......... ...C.C.... -......... .....T.... ....A..G.. ....T..AG. C..GAG..AC GTTTA.A.G. T.AG...T.. T.....T.G. ...GTAC.T. [600]

PCo ...A...... C......... ...C.C.... T......... ......TC.. .......G.. ....T..AG. C....A.CCG TT.--.A.GT T......T.. C.....C.GG .--GGAA.T. [600]

Pst ...A...... C......... ...C.C.... -......-.. ......T... .......G.. ....T..AG. C....G..AC GTTTA.A.G. T.AG...T.. T.......T. .C.GTAC.T. [600]

Pol ...A...... C......... ...C.C.... -......C.T .....T.... .......G.. ....T.TAG. CG.TAG..A. .TTCA...T. -.A...-T.. C.....GTG. AA-GTAA.T. [600]

Pma .......T.. .....C.A.A -..C...... T....C.... ....G.TG.T .......... ....G.AGGG C--TTG.TA. CCT--.A.GT G.A....TCC T..GAATTCA .--GAAA.T. [600]

CR1 AGGTGAACGG AGGGAGGATA ATGACTGAAG ATTAACAT-A AATGATATCA AGGACATAAG AAATTGAACA AATTTACTTA TATCAAATGA TATCATTTAG GATTTAAACG TTTAATAGAC [720]

CR2 .......... .......... .......... ........-. .......... .......... .......... .......... .......... .......... .......... .......... [720]

Hhi GTA.CCGT.T .AT...TC.T -.ATTA.... GA......T. .G..T..... T.TG.....A G.-------- --...G..C. .T..TTC..T C...CCCAG. ATACCCCCTT ...T--GCG. [720]

Hst GTA.CC.T.T ......TC.T -.ATTAA..A .A......T. .G..T..... T.TG.....A G.-------- --...G..C. .T..TTC.AT C...CCCAG. ATACCCCCTT ...TT-GCG. [720]

Rhi GTA.CC.T.T TATA..TC.T -.ATTA.... GA......A. .GG....... T.TG...... ..-------- --.G.G..CC .T..TTC.AT C...CCCAG. ATACCCCCTT ...T---CG. [720]

Vmo GTA.TC.T.T .ATA..TC.T -.ATTA.... .A......T. .GG....... T.TG.....A G.-------- --.A.G..C. .T.ATTC..C CT..CCCAG. ATACCCCCTT ...T--GCG. [720]

Vva GTA.CC.T.T .ACA..TC.T -.ATTA.... .A......T. ..G....... T.TG.....A ..-------- --.A.G..C. .T.ATCT..T CT..CCCAG. ATACCCCCTT ...C--GCG. [720]

PCo GTA.CC.T.A TA-ATA..CT T.ATTA.... GA..C...A. .G...A.... ..TG...... T.-------- --.G.C...G .T..TCC.AT CT..CC.AGA .T.GCCCCG. G..TTCGCG. [720]

Pst GTA.CC.T.T .AC..AAC.T -.ATTA.... .ACC....T. .TA....... ..TG...... G.-------- --.G.G...G .T.ATTC.AC CT..CCCAG. ATGCCCCCTT ...TGCGCG. [720]

Pol GTC.C..T.T .ATT.ATC.T -.AT..A... .A......T. ..A....... ..TG...... GG-------- --.GGT...G .TAATCCAA. G...CC.... ATCACCCC.T ...T--GCG. [720]

Pma GTA.T..T.A TCTAGA.GCT T.CGACA... .ACC....A. C-....T... C.AG...... -G-------- --.GCTAC.G CT..TCC.AC G.CTCC.A.. AT.ACCCC.T -..TT.GCG. [720]

CSB-2 CSB-3

CR1 GTAAACCCCC CCTACCCCCC CAAAAATACC AAACCTTTT- --ATTTCCTG AACCCCCCCA GAAACAGGAC CAGGCCTTAG TATT [804]

CR2 .......... .......... .......... .........- --........ .......... .......... .......... .... [804]

Hhi .C...A.... ..-TA..... .T...CCC.T G..GT.GC.A AG.CCC.TGA ..A......G .......... A.AC.TC.G. ..GC [804]

Hst .A...A.... ..-TA..... .T...CCC.T G..GT.GC.A AG.CCC.TGA ..A......G .......... A.AC.TC.G. ..GC [804]

Rhi .....A.... ..-TA..... .T...C.C.T G..GT.GC.A AG.CCC.TGA ..A......G .......... A.AC.TC.G. ..GC [804]

Vmo .....A.... ..CTA..... .C...C.C.T G..GT.GC.A AC.C.C.TGA ..A......G .......... A.AC.TC... ..GC [804]

Vva .....A.... ..CTA..... .T...C.C.T G..GT.GC.A AC.C.C.TGA ..A......G .......... A.AC.TC..A ..GC [804]

PCo .-...AT... ..CTA..... .....C.CGT G..GT.G..A TG..CCTGAA .........G .........- A.ACTTC... ..A. [804]

Pst .A..GA.... ..-TA..... .T.C.CCC.T G..GT.GC.A AG.CCC.TGA ..A.....-G .......... A.ACTTC.G. ..GC [804]

Pol .C...A.... ..CCA..... .-...C.C.T ..GGT.A.CT AT.C.C.TGA ..A......G .........A A.CC..GA.. C..C [804]

Pma .G...A.... ..CTA..... .CT..C.C.T ..GGT.GC.A AC.C.C.TGA ..A......G .........- A..C..C... ..G. [804]

*CR1: CR1 in *S. Latus* mitogenome; CR2: CR2 in *S. Latus* mitogenome; Hhi: *Hippoglossus hippoglossus*; Hst: *Hippoglossus stenolepis*; Rhi: *Reinhardtius hippoglossoides*; Vmo: *Verasper moseri*; Vva: *Verasper variegatus*; PCo: *Pleuronichthys cornutus*; Pst: *Platichthys stellatus*; Pol: *Paralichthys olivaceus*; Pma: *Psetta maxima*
